# Development and evaluation of an *in-house* single step loop-mediated isothermal amplification (SS-LAMP) assay for the detection of *Mycobacterium tuberculosis* complex in sputum samples from Moroccan patients

**DOI:** 10.1186/s12879-016-1864-9

**Published:** 2016-09-27

**Authors:** El Mehdi Bentaleb, Mohammed Abid, My Driss El Messaoudi, Brahim Lakssir, El Mostafa Ressami, Saaïd Amzazi, Hassan Sefrioui, Hassan Ait Benhassou

**Affiliations:** 1 Moroccan Foundation for Advanced Science, Innovation and Research (MAScIR), Rabat Design Center, Avenue Mohamed El Jazouli - Madinat Al Irfane, 10100 Rabat, Morocco; 2 Laboratory of Mycobacteria Genetics, Pasteur Institute of Morocco, Tangier, Morocco; 3 Laboratory of Tuberculosis, Pasteur Institute of Morocco, Casablanca, Morocco; 4 Laboratory of Biochemistry and Immunology, Faculty of Sciences, Mohammed V University, Rabat, Morocco

**Keywords:** Tuberculosis, Diagnosis, SS-LAMP, IS6110, MTBC

## Abstract

**Background:**

Tuberculosis (TB) is a major global health problem and remains the leading cause of morbidity and mortality in developing countries. Routinely used TB diagnostic methods, in most endemic areas, are time-consuming, often less-sensitive, expensive and inaccessible to most patients. Therefore, there is an urgent need for the development of early, easy to use and effective diagnosis tools of TB, which can be effectively integrated into resource limited settings, to anticipate the early treatment and limit further spread of the disease.

Over the last decade, Loop-mediated isothermal amplification (LAMP) assays have become a powerful tool for rapid diagnosis of infectious diseases because of the simplicity of device requirements. Indeed, LAMP is a simple, quick and cost effective Isothermal Nucleic Acid Amplification diagnostic test (INAAT) that has the potential to be used in TB endemic settings of resource-poor countries.

**Methods:**

In the present study, we have developed a simple and rapid TB molecular diagnostic test using a Single-Step Loop-mediated isothermal DNA amplification (SS-LAMP) method for the detection of *Mycobacterium tuberculosis* complex (MTBC) strains, with a simplified sample preparation procedure, eliminating DNA extraction prior to LAMP amplification, DNA initial denaturation and enzymatic inactivation steps during the amplification process.

To perform our *in-house* SS-LAMP assay, a set of six specific primers was specifically designed to recognize eight distinct regions on the MTBC species-specific repetitive insertion sequence 6110 (IS6110). The amplification of the targeted DNA was carried out under isothermal conditions at 65 °C within 1 h. Our protocol was firstly optimized using 60 of confirmed MTBC isolates and a recombinant pGEMeasy-IS6110 vector for sensitivity testing. Thereafter, the assay was evaluated on liquefied sputum specimens collected from 157 Moroccan patients suspected of having TB.

**Results:**

Our SS-LAMP developed assay was able to detect MTBC DNA directly from liquefied sputum samples without any prior DNA extraction, denaturation nor the final enzymatic inactivation step. When compared to routinely used Löwenstein Jensen (LJ) Culture method, our SS-LAMP assay is rapid and showed specificity and sensitivity of 99.14 % and 82.93 % respectively which are within the international standards. In addition, the limit of detection of our assay was found to be as little as 10 copies of bacterial DNA.

**Conclusion:**

To our knowledge, this is the first study using a single step LAMP (SS-LAMP) procedure as a rapid, easy to perform and cost effective testing for TB early detection. This innovative assay could be suitable for low-income countries with restricted health equipment facilities.

## Background

Caused by bacteria of the *Mycobacterium tuberculosis complex* (MTBC), Tuberculosis (TB) is one of the most deadly infectious diseases worldwide [1]. In 2014, there were an estimated 9.6 million incident cases of TB globally, and approximately 1.5 million people died of the disease, mainly in developing countries [2]. In Morocco, despite significant efforts to prevent, control and ensure effective management of TB, its incidence remains high with 28,135 diagnosed new cases including 14,738 of pulmonary new cases bacteriologically confirmed or clinically diagnosed, and 13 397 new cases of extra-pulmonary TB forms [2].

Early diagnosis of TB facilitates appropriate treatment initiation and can limit the spread of this highly contagious disease. Unfortunately, routinely used TB diagnostic methods are time consuming, often less sensitive, and inaccessible to most of patients in endemic countries that lack necessary and appropriate financial and human resources.

In Morocco, as in low and middle income countries, the World Health Organization (WHO) and the International Union Against Tuberculosis and Lung Disease (IUATLD) recommend the diagnosis of TB by sputum smear microscopy as a crucial and inevitable step in testing patients suspected of having pulmonary TB [2, 3]. This technique seems to be simple, rapid and inexpensive but has the inconvenience to be less sensitive and requires repeated patient’s sputum examinations [3].

Otherwise, conventional culture-based methods, because of their high specificity remain the gold standard test for the diagnosis of pulmonary TB in Morocco reference laboratories. Despite its outstanding performance (100 times more sensitive than smear microscopy), the mycobacterial culture is notoriously known as time-consuming, dangerous and costly in terms of investment in infrastructure and equipment [4–6].

When compared to TB routing testing assays, Nucleic Acid Amplification Tests (NAATs) are most promising and more relevant in terms of sensitivity and rapidity. However, expensive infrastructure and advanced technical skills requirements to conduct these tests are beyond the scope of most diagnostic facilities offering TB diagnostics to communities, particularly in high TB-burden countries where the majority of TB cases occur [7–10].

While most of TB diagnostic NAATs use RT-qPCR technology, which needs precision, thermal cycler and sophisticated optical detection systems that add complexity and cost to the instrumentation [11], Loop-mediated Isothermal Amplification (LAMP) remains an accessible and cost-effective alternative assay to rapidly detect and type mycobacteria in pulmonary samples. Indeed, LAMP requires just constant incubation temperature, and therefore can be implemented with simplified instrumentation [12–16].

In the present study, we established for the first time a simple and rapid single step LAMP (SS-LAMP) assay without DNA extraction and denaturation and without enzyme inactivation steps for the detection of MTBC strains. We also compared sensitivity, specificity and applicability of our *in-house* SS-LAMP assay to LJ culture, as the Gold Standard routine testing.

## Methods

### Clinical samples

This study was performed conforming to the ethics charter of Pasteur Institute (Morocco) that approved the consent procedure and the proposals of the used protocols. The protection of patient’s clinical information and preservation of the results confidentiality were guaranteed in the framework of that charter. According to conventional WHO guidelines, a total of 60 culture positive isolates and 157 sputum were collected at Pasteur Institute from Moroccan patients suspected of having pulmonary TB.

### SS-LAMP primers design

**Fig. 1:**
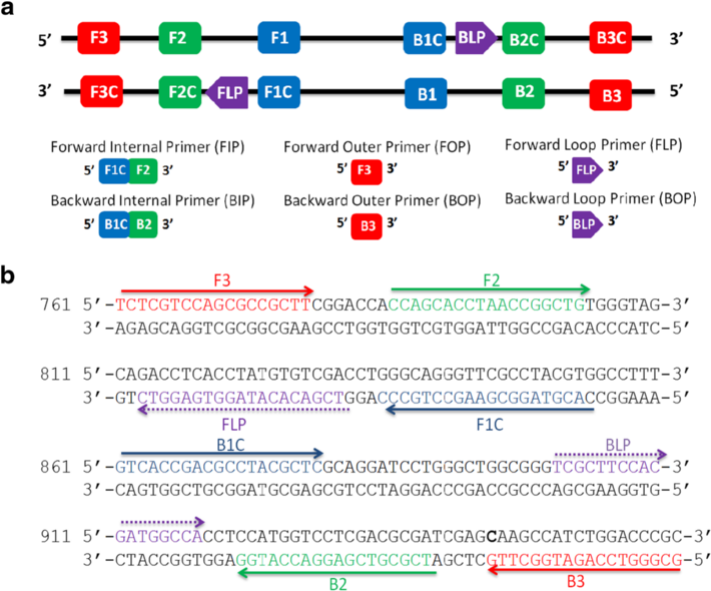
Location and sequence of primers. **a** Location and sequence of SS-LAMP target and priming sites. **b** Annealing position and amplification orientation of the SS-LAMP primers on the selected 234-bp target sequence of the IS6110

A set of SS-LAMP primers complementary to the nucleotide sequence of IS6110 (GenBank accession number: X17348) was designed using the online Primer Explorer V4 software (http://primerexplorer.jp/elamp4.0.0/index.html; Eiken Chemical Co., Ltd., Tokyo, Japan) according to the consensual criteria as described by Notomi and collaborators [17]. IS6110 specific SS-LAMP primers were specifically chosen to recognize eight distinct regions on the target gene: forward inner primer IS-FIP (F1c-F2), backward inner primer IS-BIP (B1c-B2), and two outer primers, IS-FOP (F3) and IS-BOP (B3). Two loop primers IS-FLP and IS-BLP used to accelerate the reaction by providing more starting sites for the auto-cycling amplification were designed manually [13, 16]. The directions and details of the primers sequences are shown in (Fig. 1, Table 1).

**Table 1:** Nucleotide sequences of the SS-LAMP primers targeting the IS6110 sequence of MTBC strains

Primer type	Positions	Sequence 5’-3’
FIP(F1c-F2)	835-853/786-803	ACGTAGGCGAACCCTGCCCCCAGCACCTAACCGGCTG
BIP(B1c-B2)	861-879/922-939	GTCACCGACGCCTACGCTCTCGCGTCGAGGACCATGG
F3 (FOP)	761-778	TCTCGTCCAGCGCCGCTT
B3 (BOP)	945-962	GCGGGTCCAGATGGCTTG
FLP	813-831	TCGACACATAGGTGAGGTC
BLP	901-918	TCGCTTCCACGATGGCCA

These primers were assessed for specificity before use in LAMP assays by using the Basic Local Alignment Search Tool (BLAST) with sequences in GenBank of the National Centre for Biotechnology Information (NCBI). All the primers were synthesized by Eurofins (Eurofins MWG Operon, France) and purified using high performance liquid chromatography (HPLC).

### Reference plasmid

To assess the sensitivity of our *in-house* SS-LAMP assay, a recombinant plasmid containing the target sequence of IS6110 (Genbank Accession Number: X173) was constructed. The Genomic DNA of *Mycobacterium tuberculosis H37Ra* (ATCC:25177D-5, USA) was used to amplify a 202 bp fragment located between the F3 and B3 primer binding site of IS6110 targeted region by using the IS-F (IS-FOP) and IS-R (IS-BOP) primers. PCR reactions was performed on Veriti PCR instrument (Applied Biosystems, USA) under the following cycling conditions: (i) an initial step at 95 °C for 5 min, (ii) 35 cycles at 95 °C for 45 s, 58 °C for 45 s and 72 °C for 45 s and (iii) a final elongation at 72 °C for 7 min. The PCR products were checked by agarose gel (1 %) electrophoresis, purified with a Wizard SV gel and PCR clean up system (Promega, USA) and then cloned into pGEM-T Easy Vector (Promega, USA). The positive clones obtained after transformation of competent *E. coli DH5-alpha* strain (New England Biolabs, USA) were then checked out by PCR and the resulting amplicons were identified at the expected size. The obtained recombinant plasmid was quantified by NanoDrop 2000 (Thermo Scientific, USA) and serially diluted to concentrations of 10^6^, 10^5^, 10^4^, 10^3^, 10^2^, 10 and 1 copy/reaction. The dilutions were used to evaluate the limit of detection (LOD) of the SS-LAMP assay and the reference plasmid as a positive control for further SS-LAMP reactions.

### SS-LAMP reaction

Our *in-house* SS-LAMP assay was set up in a final volume of 25 μl based on the original LAMP method described previously by Notomi et al. [17] with some interesting modifications allowing us to save time and costs of the reaction. Indeed, the reaction volume contained 1.6 μM for IS-FIP and IS-BIP primers, 0.8 μM for loop primers (IS-FLP and IS-BLP), 0.2 μM for IS-FOP (F3) and IS-BOP (B3) outer primers, 2 mM of dNTPs, 0.8 M Betaine (Sigma-Aldrich, USA), 20 Mm Tris-HCl (pH 8.8), 10 mM KCl, 10 mM (NH4)_2_SO4, 9 mM MgSO4, 0.1 % Triton X-100, and 8 U of *Bst 2.0 WarmStart DNA Polymerase* (New England Biolabs, USA), along with 5 μl of DNA template. Furthermore, in order to determine whether LAMP can be performed under isothermal conditions at all steps, we attempted to perform the reactions without an initial heat-denaturation of template DNA and also by removing the final enzyme inactivation step. Indeed, all the reactions within a final volume of 25 μl were directly incubated for a single step of amplification at 65 °C during 60 min.

**Fig. 2:**
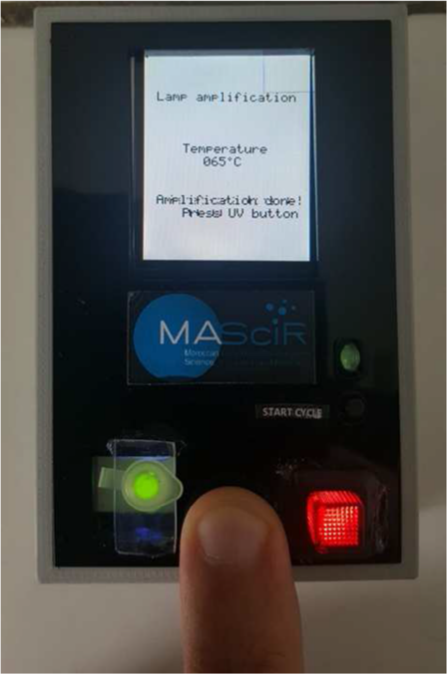
SS-LAMP on a Lab-on-card device. Prototype of our *in house* Lab-on-card device (LOC) and a visual detection of SS-LAMP reaction performed on this prototype

Moreover and in order to assess the usefulness and efficacy of different heating devices on SS-LAMP amplification, a thermal cycler, a water bath, a dry thermal block with a 0.2-mL PCR tube holder and finally a Lab-on-card prototype (Fig. 2), were used to maintain the temperature required for the amplification reaction. To measure constantly the water bath and the dry thermal block temperatures during the experiments, a calibrated thermometer was used. However, for the thermal cycler (calibrated every six months) and the *in-house* Lab-on-card device, an integrated electronic circuit allowed a real-time verification of the heating bloc and the Peltier System temperatures respectively.

A negative control (DEPC-treated water) and a positive control (Genomic DNA of *M. tuberculosis H37Ra* reference strain) (ATCC, Manassas, VA, USA) were used for every run.

In our present study, the results were visualized by adding 1 μl of 1:10 SYBR Green I stock dilution ($US 0.08/reaction) (Life Technologies, USA) and then, observed under both visible light and UV light. A yellowish green colour (under natural light conditions) indicated a positive reaction, while orange indicated a negative reaction. SS-LAMP products were then subjected to 2 % agarose gel electrophoresis, stained by EZ-Vision® DNA Dye (Amresco, USA), and visualized under UV light. All the SS-LAMP reactions have been performed in triplicate to assess to reproducibility of the assay.

### Determination of the primers specificity and limit of detection of the SS-LAMP assay

To verify their specificity for the detection of MTBC IS6110 sequence, our selected primers were tested using DNA from 60 MTBC positive isolates provided by the Pasteur Institute of Morocco. These MTBC isolates were previously confirmed by conventional PCR and LJ culture method. SS-LAMP primers have been also tested on some MTBC strains DNA (*M. tuberculosis H37Rv,* ATCC 25618D-2; *M. tuberculosis H37Ra,* ATCC 25177D-5*; M. bovis,* ATCC-BAA-*935D-2*) and non-MTBC strains DNA (*M. avium,* ATCC-BAA-968D-5*; M. kansasii, ATCC 12478; M. intracellulare, ATCC 35769; M. abscessus, ATCC 19977D-5; M. gordonae, ATCC 14470D-5; M. smegmatis, ATCC 700084D-5; Escherichia coli, ATCC 8739; Pseudomonas aeruginosa, ATCC 9027; Staphylococcus aureus, ATCC 6538; Candida albicans, ATCC 10231; Aspergillus niger, ATCC 16888*).

To explore the sensitivity, the limit of detection (LOD) of our SS-LAMP assay was assessed by serial dilutions of the purified reference plasmid (pGEMeasy-IS6110) with DEPC-treated water to obtain a standard series from 10^6^ to 1 copy per reaction. The serial dilutions were subjected to amplification reaction conditions exactly as previously described. All the reactions were performed in 3 replicates. *Escherichia coli* bacteria DNA and DEPC-treated water were used as negative controls.

### SS-LAMP assay on clinical sputum samples

Once optimized, the SS-LAMP assay was performed on clinical sputum specimens to evaluate the feasibility and accuracy of the assay when compared to culture isolate results. For that purpose, 157 sputum samples from TB suspected patients, collected by the Pasteur Institute were liquefied and decontaminated using 4 % NaOH. A part of the liquefied sputum was used for culture in LJ solid medium following the standard protocol and a second part directly utilized for SS-LAMP evaluation. In the present study, SS-LAMP reactions were performed using 5 μl of liquefied/decontaminated sputum without any prior heat treatment, DNA extraction procedure or kits. To control any eventual cross-contamination, one negative control was also used for every run.

### Statistical analysis

Sensitivity and specificity [18] as well as the positive and negative likelihood ratios [19] of the SS-LAMP assay were calculated in comparison with the standard LJ culture results.

## Results

### Optimization of the SS-LAMP assay using different amplification devices

In the present study, 3 sets of primers, specific to MTBC IS6110 sequence, were used for the SS-LAMP amplification. The set that showed the highest amplification performance was selected for further analysis (Fig. 1, Table 1). To optimize the SS-LAMP reaction temperature, we conducted the assay under two different temperature conditions (63 °C and 65 °C). Given the important amount of DNA amplicons produced on both thermal conditions and based on the optimal temperature for Bst 2.0 *WarmStart DNA Polymerase* activity, an incubation temperature of 65 °C was used for all tests in this study.

In order to determine whether our SS-LAMP assay can be performed in a single step procedure, we amplified the IS6110 target sequence directly from the liquefied sputum samples. Indeed, reactions mixtures were carried within a final volume of 25 μl without any prior DNA extraction, initial DNA heat denaturation nor enzymatic inactivation.

**Fig. 3:**
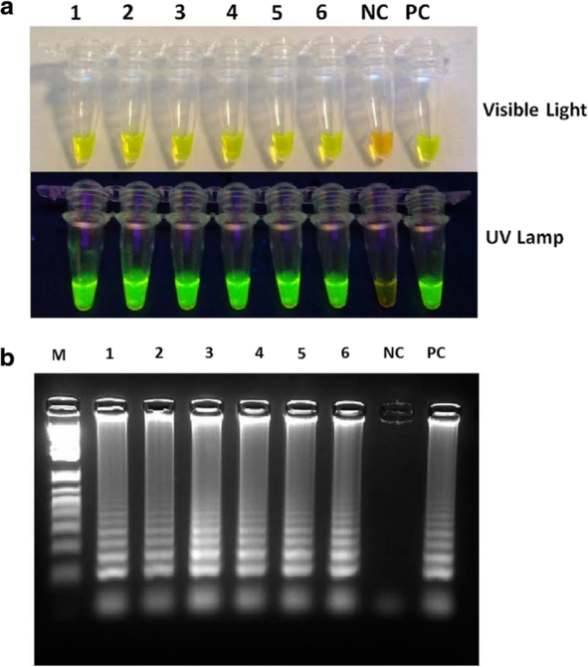
Detection of SS-LAMP. **a** Visual Detection of SS-LAMP products using inspection of the color change in 1000 X SYBR Green I by the naked eye under visible and UV lights respectively: Samples that turned yellowish green were considered positive, while those remained orange were assumed to be negative. Tube 1 to 6: SS-LAMP products of *Mycobacterium tuberculosis* extracted genomic DNA. NC: Negative Control. PC: Positive Control (*M. tuberculosis H37Ra* genomic DNA). **b** Lanes 1 to 6: Gel electrophoresis of SS-LAMP products of *Mycobacterium tuberculosis* genomic DNA. NC: Negative Control. PC: Positive Control (*M. tuberculosis H37Ra* genomic DNA). M: 1 kb Ladder

DNA amplification was completed within one hour at isothermal condition (65 °C in a water bath). The obtained products of the SS-LAMP reactions were then detected by SYBR Green nucleic acid stain. Positive samples changed colour from orange to green while negatives remained orange (Fig. 3a). The products were also viewed on a 2 % agarose gel and showed ladder-like patterns (Fig. 3b). The same results have been obtained when the SS-LAMP amplification was performed on other heating devices, rather than the water bath, such as a thermal cycler and a dry thermal block (data not shown).

Presently, we are developing an innovative temperature controlled Lab-on-card prototype based on our SS-LAMP method, described in the present study (Fig. 2). This portable battery powered device, conceived to be used in remote low-resource settings without electricity, showed encouraging preliminary data, similar to the ones obtained using water bath (Fig. 3a). Thus, MTBC positive samples turned to green under UV excitation whereas the negative control remained orange.

### Specificity of the SS-LAMP assay primers

First of all, we examined the specificity of our selected SS-LAMP primers using genomic DNA from 60 previously confirmed MTBC isolates by Gold Standard culture and conventional PCR. Efficient DNA amplification was assessed using *M. tuberculosis complex* including *M. tuberculosis H37Rv, M. tuberculosis H37Ra and M. bovis*. The results showed that no DNA amplifications were observed when using non-MTBC bacterial species (data not shown). These results confirm that our selected SS-LAMP primers are highly specific to MTBC strains.

### Limit of detection of the SS-LAMP assay

**Fig. 4:**
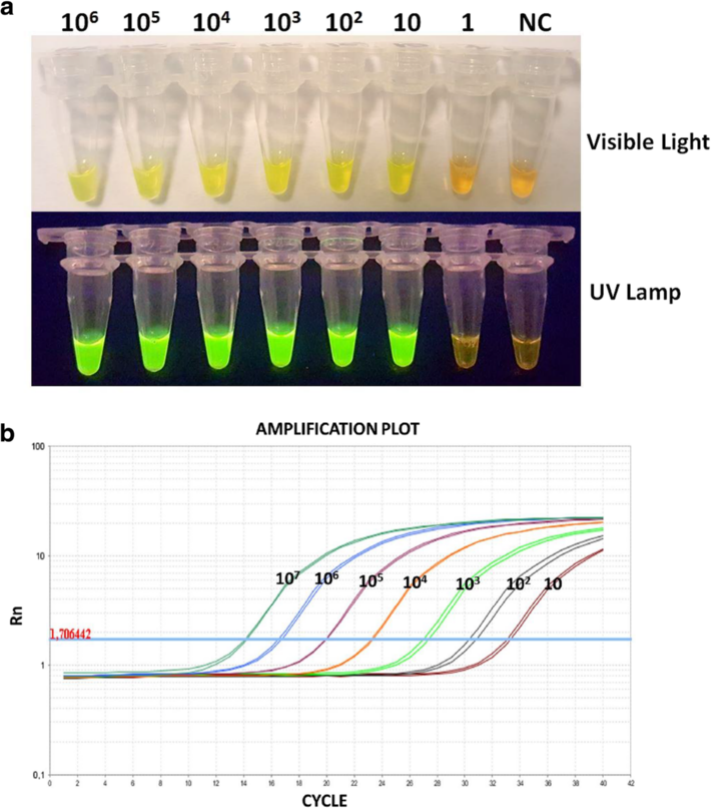
Limit of detection (LOD) of SS-LAMP. **a** Limit of Detection (LOD) of the SS-LAMP assay. Tubes 1–7 showed the color change from original orange color to bright green. The tubes contained 10^6^, 10^5^, 10^4^, 10^3^, 10^2^, 10 and 1 copy/reaction of purified reference plasmid (pGEMeasy-IS6110), respectively. NC: Negative control. **b** Limit of Detection (LOD) of RT-qPCR reaction: Amplification plot shows the normalized fluorescent signal (Rn) versus cycle number. The reaction wells contained duplicates of 10^6^, 10^5^, 10^4^, 10^3^, 10^2^, 10 and 1 copy/reaction of purified reference plasmid (pGEMeasy-IS6110), respectively

The sensitivity of the SS-LAMP assay for MTBC strains was assessed by the limit of detection (LOD) method using serial 10-fold dilutions of reference plasmid pGEMeasy-IS6110, as previously described. The results indicated that the LOD of the SS-LAMP assay was 10 copies of IS6110 sequence (Fig. 4). This SS-LAMP LOD was comparable to the one of RT-qPCR, performed in our previous studies [20, 21] on reference plasmid pGEMeasy-IS6110 (Fig. 4).

### Clinical comparative study between SS-LAMP and Löwenstein-Jensen culture method

The clinical results of a total of 157 sputum samples were determined using both the SS-LAMP assay and the LJ culture method. Briefly, thirty four patient sputum samples used in this study were positive in LJ culture and SS-LAMP method and 115 specimens were negative in LJ culture as well as in SS-LAMP. Seven sputum that were tested MTBC negative using SS-LAMP, grew on the LJ solid medium. Finally, for one sputum sample, the SS-LAMP was positive and the LJ culture negative.

The analysis of these results using reference methods [18, 19] demonstrated that SS-LAMP showed good sensitivity (82.3 %) and specificity (99.14 %) for the used clinical sputum specimens compared to culture (*n*  =  157), as shown in Table 2. Accordantly, the positive likelihood ratio and the negative likelihood ratio were 96.20 and 0.17 respectively (Table 2). Hence, these results indicate that our SS-LAMP assay described here could be efficiently applied for TB diagnosis by testing directly patient sputum samples.

**Table 2:** Efficacy of SS-LAMP assay compared to gold standard LJ culture

	LJ Culture		Sensitivity %	Specificity %	Negative likelihood ratio	Positive likelihood ratio
	Positive	Negative				
LAMP (*n* = 157)			82.93	99.14	0.17	96.20
Positive	34	1				
Negative	7	115				
	%95CI =		67.94 - 92.85	95.29 – 99.98	0.09 - 0.34	13.60 - 680.51

## Discussion

Loop-mediated isothermal amplification (LAMP) is an innovative method that has been used recently at the basis of the development of a number of diagnostic methods to detect several infectious diseases including TB.

The present study aims to develop and evaluate a novel *in-house* LAMP-based procedure, so called single step LAMP (SS-LAMP), for the detection of *M. tuberculosis complex* strains in clinical sputum samples. For that purpose, we explored whether we can perform the classical LAMP method in one uninterrupted process, with no need to any previous DNA extraction, denaturation nor final enzymatic inactivation step. Hence, when compared to classical LAMP, the SS-LAMP is time and cost-effective and could be a suitable alternative method for TB diagnosis in low income developing countries.

Before the optimization of our SS-LAMP assay, we first demonstrated the high specificity of our selected primer set, using 60 separate cultures isolates obtained from the sputum of TB positive patients. Secondly, a limit of detection (LOD) of 10 copies per reaction showed the high sensitivity of our SS-LAMP assay which was equal to the LOD of the highly sensitive RT-qPCR method (Fig. 4).

To set-up the optimization of our SS-LAMP procedure, a *WarmStart Bst DNA Polymerase*, active relatively at high isothermal amplification temperature, reducing non-specific priming, was used. The SS-LAMP assay was optimized in a way allowing sensitive detection of all MTBC strains within a 60 min reaction, in culture isolates or sputum samples.

The SS-LAMP starts with the direct mixing, in a PCR tube, of a small volume of liquefied and decontaminated patient’s sputum added to the reaction master mix. The latter is then incubated directly at 65 °C during one hour. The only equipment needed for this assay is a water bath or a heat block that furnishes a constant temperature of 65 °C. A reference plasmid, pGEMeasy-IS6110, was constructed to be included into the SS-LAMP assay as a positive control instead of using a genomic DNA of Mycobacterium references strains whose the preparation is expensive and time consuming.

To assess the correlation between the results obtained from the SS-LAMP and the Gold Standard LJ culture method, a total of 157 collected patient’s sputum were liquefied and decontaminated prior to both culture and SS-LAMP evaluation. After LJ medium inoculation, the small remaining volume of decontaminated sputum was then used for SS-LAMP testing. The clinical comparison of the results was performed using the analytical specificity and sensitivity methods as described in the literature [18, 19]. As shown in table 2, when compared to LJ culture, our SS-LAMP showed high specificity (99.14 %) and sensitivity (82.93 %). These results are much higher to the one described in the literature for the MTBC detection by smear microscopy which showed up to 60 % maximum sensitivity when compared LJ culture [22]. Interestingly, the specificity and sensitivity of our SS-LAMP when compared to LJ culture are within the range of those reported earlier using the original LAMP method performed on extracted DNA samples and involving DNA denaturation and enzyme inactivation steps [23]. In fact, a LAMP assay, performed on lysed samples and in 2 h amplification reaction showed a sensitivity and specificity values of 79.5 % and 93.8 % respectively, which are remarkably much lower than the obtained values of the present study [23]. The unique specimen that was negative on LJ culture and LAMP positive (Table 2) may suggest that SS-LAMP is probably highly sensitive in the detection of a small number of tubercle bacilli released from hidden foci of this patient sputum. SS-LAMP might also detect MTBC circulating DNA from non-viable lysed bacteria and hence non-culturable on LJ medium.

Otherwise, seven specimens that were positive on LJ culture were SS-LAMP negative (Table 2). This discordance could be due to false positive cultures of *Mycobacterium tuberculosis* that resulted from laboratory cross-contamination. The presumed false positive cultures in our study represent a rate of 4.45 % (7 of 157) which is similar to the rates shown in previous studies of *M. tuberculosis* cultures [24–26]. In fact, in most population-based studies, rates of false positive cultures has been reported to be ranging from 2.2 – 10.5 % [27]. Indeed, many causes can lead to false-positive cultures such as clerical errors, contaminated clinical equipment, laboratory cross-contamination, contamination from species identification procedures [26] or a faulty exhaust hood [28]. Furthermore, the results discrepancies between LJ culture and SS-LAMP can also be caused by the presence into the patient’s specimens of nontuberculous mycobacteria that can easily grow on LJ culture medium [29]. Otherwise, MTBC strains lacking the IS6110 insertion sequence might explain the seven missed samples [30]. In fact, these strains may escape detection by our SS-LAMP assay that specifically target the IS6110 sequence. Unfortunately, we do not have yet sufficient data on the prevalence of these MTBC strains in Morocco and in neighboring countries. To date only a single study has evaluated the frequency of *M. tuberculosis* strains Lacking IS6110 among 102 sputa isolated from Moroccan patients and have shown that only two *M. tuberculosis* strains were lacking the insertion sequence [31]. In all circumstances, to overcome this limitation, including in our assay a complementary MTBC-specific gene such as MPB64 will allow certainly to cover the whole MTBC strains spectrum.

Overall, it is actually well known that the most miss-advantageous troubleshooting of the LAMP methodology consists of getting false positive result which is, basically, due to aerosol cross-contamination between tubes when the dye was added to the reaction [32, 33]. These studies have demonstrated that the high risk of contamination with LAMP is due, essentially to the exposure of tubes to the high temperature during enzyme inactivation. This final step was removed from our patented SS-LAMP procedure [20, 21] that has proven its undeniable capacity to be efficient, specific and reproducible in the detection of the target under a constant temperature, which allows overcoming the possible cross-contamination that may result from the enzymatic inactivation of previous reactions. In addition, in our study, we proved that the SS-LAMP assay can keep the noteworthy features to include just a heat block, a simple water bath or a cost effective Lab-on-card device instead of a costly thermal cycler, gel electrophoresis system and UV trans-illuminator. Furthermore, our SS-LAMP assay is performed under isothermal conditions at a constant temperature of 65 °C, circumventing the time length involved in thermal changes of conventional amplification methods.

## Conclusion

In conclusion, the SS-LAMP developed in this study provides a number of advantages over microscopy and culture including superior sensitivity and significantly less operating time. Furthermore, the SS-LAMP constitutes an easily handled, one step diagnostic tool for rapid, sensitive, specific and accurate identification of *M. tuberculosis* complex, in both culture isolates and sputum specimens. Thanks to its simplicity, robustness, easy use without necessary sophisticated equipment and its feature to be performed in simple laboratories, small-scale hospitals and primary care facilities in developing countries, the SS-LAMP promotes to be suitable for diagnostic of TB as a point of care (POC) assay.

## Abbreviations

INAAT: Isothermal nucleic acid Amplifications diagnostic test
IS6110: Insertion sequence 6110
LAMP: Loop-mediated isothermal amplification
LJ: Löwenstein Jensen
LOC: Lab-on-card
LOD: Limit of detection
MTBC: *Mycobacterium tuberculosis* complex
NAATs: Nucleic acid amplification tests
PCR: Polymerase chain reaction
POC: Point of care
RT-qPCR: Real time quantitative polymerase chain reaction
SS-LAMP: Single-Step Loop-Mediated Isothermal Amplification
TB: Tuberculosis
